# Engineering 3D Heterostructured NiCo_2_S_4_/Co_9_S_8_-CNFs via Electrospinning and Hydrothermal Strategies for Efficient Bifunctional Energy Conversion

**DOI:** 10.3390/nano15201559

**Published:** 2025-10-13

**Authors:** Dhananjaya Merum, Rama Krishna Chava, Misook Kang

**Affiliations:** Department of Chemistry, College of Natural Sciences, Yeungnam University, 280 Daehak-Ro, Gyeongsan 38541, Gyeongbuk, Republic of Korea; msdhana@yu.ac.kr (D.M.)

**Keywords:** NiCo_2_S_4_, carbon nanofibers, core-shell nanostructure, oxygen evolution, methanol oxidation, bifunctional catalyst

## Abstract

The rational design of multifunctional electrocatalysts requires synergistic integration of conductive scaffolds with redox-active components. Here, a hierarchical core–shell NiCo_2_S_4_ grown/anchored on Co_9_S_8_-loaded carbon nanofibers (NCS/CS/CNFs) was synthesized via an electrospinning and hydrothermal approach and systematically characterized. FESEM/TEM confirmed a core-shell nanofiber structure with a NiCo_2_S_4_ shell thickness of ~30–70 nm, increasing the fiber diameter to ~290 ± 30 nm, while BET analysis revealed a surface area of 24.84 m^2^ g^−1^ and pore volume of 0.042 cm^3^ g^−1^, surpassing CS/CNFs (6.12 m^2^ g^−1^) and NCS (4.85 m^2^ g^−1^). XRD confirmed crystalline NiCo_2_S_4_ and Co_9_S_8_ phases, while XPS identified mixed Ni^2+^/Ni^3+^ and Co^2+^/Co^3+^ states with strong Ni-S/Co-S bonding, indicating enhanced electron delocalization. Electrochemical measurements in 1 M KOH demonstrated outstanding OER activity, with NCS/CS/CNFs requiring only 324 mV overpotential at 10 mA cm^−2^, a Tafel slope of 125.7 mV dec^−1^, and low charge-transfer resistance (0.33 Ω cm^2^). They also achieved a high areal capacitance of 1412.5 μF cm^−2^ and maintained a stable current density for >5 h. For methanol oxidation, the composite delivered 150 mA cm^−2^ at 0.1 M methanol, ~1.6 times that of CS and 1.3 times that of NCS, while maintaining stability for 18,000 s. This bifunctional activity underscores the synergy between conductive CNFs and hierarchical sulfides, offering a scalable route to durable electrocatalysts for water splitting and direct methanol fuel cells.

## 1. Introduction

The increasing demand for sustainable energy technologies is driven by the urgent need to mitigate climate change, environmental degradation, and the depletion of fossil resources. Renewable energy sources such as solar and wind offer promising alternatives; however, their intermittency necessitates the development of efficient and scalable energy conversion and storage systems to stabilize the power supply [1,2,3,4,5]. Electrochemical devices, including water electrolyzers, metal-air batteries, and fuel cells, are desirable because they enable the direct and efficient interconversion of chemical and electrical energy with high efficiency [6]. Among these processes, the oxygen evolution reaction (OER) plays a vital role in water electrolysis and rechargeable metal-air batteries. However, it is hampered by sluggish four-electron kinetics and substantial overpotentials. In addition, the methanol oxidation reaction (MOR), central to direct methanol fuel cells (DMFCs), suffers from low catalytic efficiency, poisoning by carbonaceous intermediates, and poor long-term stability [7,8,9,10,11,12,13,14,15,16,17,18]. Noble metal catalysts, such as IrO_2_ and RuO_2_ (for OER), and Pt-based materials (for MOR) remain benchmarks; however, their scarcity, high cost, and limited durability restrict their large-scale application [19,20,21]. Hence, the development of low-cost, durable, and bifunctional electrocatalysts for both OER and MOR is of critical importance. Transition metal sulfides (TMSs), particularly Ni-Co sulfides, have emerged as attractive alternatives owing to their rich redox chemistry, tunable electronic structure, and relatively low cost [22,23,24]. Despite these advantages, conventional TMS catalysts still face limitations, including moderate conductivity, insufficient structural stability, and restricted bifunctional activity in alkaline environments [24,25].

Recent studies have shown that the catalytic performance of Ni/Co sulfides is strongly dependent on their morphology and structural configuration. For example, Co_3_O_4_@Co_9_S_8_ heterostructures grown on Ni foam achieved an OER overpotential of ~80 mV at 10 mA cm^−2^ with a Tafel slope of 107.2 mV dec^−1^, attributed to efficient charge transfer across the oxide-sulfide interface [26]. Sulfur-vacancy-engineered Co_9_S_8_ core-shell hollow spheres displayed dual activity, with an OER overpotential of 294 mV at 10 mA cm^−2^ and a MOR current density of 164.9 mA cm^−2^ at 1.8 V vs. RHE, enabled by their high surface area and optimized electronic states [27]. Similarly, core-shell Au@NiCo_2_S_4_ nanoparticles reduced the OER overpotential to 290 mV at 10 mA cm^−2^, with a Tafel slope of 44.5 mV dec^−1^, benefiting from the conductive Au core that promoted the formation of high-valence Ni/Co species [28]. Three-dimensional mushroom-like NiCo/NiCo_2_S_4_@NiCo arrays on Ni foam enabled overall water splitting at a cell voltage of 1.55 V and a current density of 10 mA cm^−2^, owing to their hierarchical architecture with abundant exposed edges [29]. Carbon-supported hybrid systems also play a crucial role in enhancing activity and durability [30]. NiCo_2_S_4_@graphene hybrids showed bifunctional activity, achieving an OER overpotential of ~350 mV and improved oxygen reduction reaction (ORR) currents. The graphene scaffold enhanced the conductivity and dispersion of active sites [31]. Similarly, Co_9_S_8_ nanoparticles embedded in N/S dual-doped hollow carbon nanofibers showed excellent durability and improved performance in terms of both ORR and OER [32]. More recently, bi-phase NiCo_2_S_4_-NiS_2_ deposited on carbon fiber paper achieved an OER overpotential of 165 mV at 10 mA cm^−2^ with a Tafel slope of 81.54 mV. dec^−1^, demonstrating the synergistic role of spinel and pyrite phases [33]. These examples underscore the importance of rational morphology control, heterostructure engineering, and conductive supports in optimizing bifunctional activity [30].

One promising strategy involves the construction of amorphous crystalline heterostructures, which leverage interfacial interaction, create defect-rich active sites, and improve charge transport [34,35,36]. In particular, embedding amorphous Co_9_S_8_ within conductive carbon nanofibers (CNFs) and coating with crystalline NiCo_2_S_4_ spinels provides a highly effective design. The CNF framework offers high conductivity and porosity [29], the amorphous Co_9_S_8_ introduces abundant defect-rich active sites [31], and the crystalline NiCo_2_S_4_ shell contributes enhance redox activity through multiple oxidation states [29,32]. The resulting amorphous/crystalline interfaces promote charge redistribution and accelerate catalytic kinetics.

Herein, we report the synthesis of a 3D NiCo_2_S_4_/Co_9_S_8_-CNF (NCS/CS/CNF) heterostructure via electrospinning and hydrothermal methods. This architecture integrates a conductive CNF network with a defect-enriched amorphous Co_9_S_8_ core and a redox-active crystalline NiCo_2_S_4_ shell. The synergistic interactions at the amorphous/crystalline interface accelerate charge transfer, enhance catalytic kinetics, and ensure long-term durability. Electrochemical characterization reveals that this heterostructure exhibits outstanding bifunctional activity in relation to both OER and MOR, along with excellent stability. Beyond introducing novel heterostructured materials, this study also establishes a generalizable design principle for hierarchical electrocatalysts in next-generation energy conversion systems.

## 2. Materials and Methods

A combination of synthesis, structural, and electrochemical methods was employed to design and evaluate Ni-Co sulfide-based carbon nanofiber composites. These approaches were selected to systematically investigate the morphology, phase composition, surface chemistry, and catalytic activity of the materials, thereby providing a comprehensive understanding of their performance in OER and MOR reactions.

### 2.1. Chemicals

All reagents were of analytical grade and used without further purification. Polyacrylonitrile (PAN, Mw = 90,000) and N,N-dimethylformamide (DMF) were used as the polymer matrix and solvent, respectively. Metal precursors, including cobalt acetate tetrahydrate (C_4_H_14_CoO_8_), nickel nitrate hexahydrate (Ni(NO_3_)_2_.6H_2_O; ≥98.0%), and cobalt nitrate hexahydrate (Co(NO_3_)_2_.6H_2_O; ≥98.0%), were used. Additional chemicals, such as thiourea (NH_2_CSNH_2_; ≥99.0%), ammonium fluoride (NH_4_F; ≥98.0%), potassium hydroxide (KOH; ≥85%), methanol (CH_3_OH; ≥99.9%), hydrochloric acid (HCl), and Nafion solution, were also utilized. Nickel foam was employed as the current collector. All chemicals were obtained from Merck (Darmstadt, Germany), Sigma-Aldrich (St. Louis, MO, USA), and the MTI Corporation (Richmond, CA, USA). Deionized water was used as the solvent medium throughout the experiments. Materials such as 20 mL syringes and aluminum foil were used during the preparation process.

### 2.2. Synthesis of CS/CNFs, NCS, and NCS/CS/CNFs

**Figure 1:**
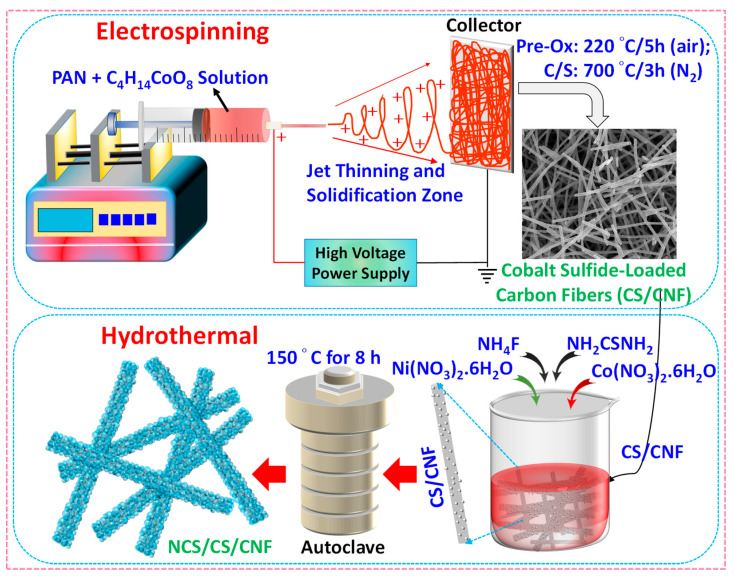
Schematic illustration of the synthesis of NCS/CS/CNF composites via electrospinning of PAN–cobalt acetate precursor, followed by carbonization/sulfurization and subsequent hydrothermal growth of Ni-Co sulfide nanostructures.

NCS/CS/CNF heterostructures were synthesized through a combined electrospinning, carbonization, and hydrothermal growth process (Figure 1). For the preparation of Co-loaded nanofibers, 2.0 g of PAN was dissolved in 20 mL of DMF under stirring for 6 h at room temperature, followed by the addition of 0.747 g of cobalt acetate tetrahydrate and continued stirring for 10 h. The resulting pink solution was loaded into a 20 mL syringe and electrospun at a voltage of 15 kV, a flow rate of 0.4 mL/h, and a tip-to-collector distance of 15 cm, using aluminum foil as the collector. The collected nanofibers were stabilized in air at 220 °C for 5 h and then carbonized as well as sulfurized at 700 °C for 3 h in a nitrogen atmosphere, using sulfur powder as the sulfur source. This treatment yielded Co_9_S_8_ nanoparticles embedded in a conductive carbon nanofiber (CS/CNF) matrix.

To fabricate NCS/CS/CNFs, 0.3 g of CS/CNFs was dispersed in 50 mL of deionized water. A 1:2 molar ratio of nickel nitrate hexahydrate (1 mM) and cobalt nitrate hexahydrate (2 mM), along with 4 mM of thiourea and 4 mM of NH_4_F, was added subsequently. After uniform dispersion, the mixture was sealed in a Teflon-lined autoclave and heated at 150 °C for 8 h. The product was collected by centrifugation, washed six times with deionized water and ethanol, and dried at 80 °C overnight. For comparison, pure NiCo_2_S_4_ (NCS) was prepared under identical hydrothermal conditions without the addition of CS/CNFs. During carbonization/sulfurization, cobalt reacts with sulfur vapor to form Co_9_S_8_, while PAN is converted into conductive carbon nanofibers. Subsequent hydrothermal treatment facilitates the nucleation and growth of NiCo_2_S_4_ on the CS/CNF scaffold, resulting in the formation of the core-shell heterostructure. The synthetic transformations can be summarized as follows:

(1) $$(C_{3}H_{3}N{)}_{n}\;+\;\mathrm{Co}({\mathrm{CH}}_{3}\mathrm{COO}{)}_{2}.4H_{2}O\overset{DMF,\;\;\;stirring}{\to }\;\text{PAN-Co}\;\mathrm{solution}$$

(2) $$\text{PAN-Co}\;\mathrm{sol}.\overset{Electrospinning\;(15\;kV,\;\;\;0.4\;mL\;h^{-1})}{\to }\;\text{Co-loaded}\;\mathrm{PAN}\;\mathrm{nanofibers}$$

(3) $$\text{Co-PAN}\;\mathrm{nanofibers}\overset{220\;{}^{\circ}C,\;\;\;air}{\to }\;\mathrm{Oxidized}\;\mathrm{Co}\;\mathrm{species}\;+\;\mathrm{stabilized}\;\mathrm{PAN}$$

(4) $$\mathrm{Stabilized}\;\text{PAN-Co}+S\;(\mathrm{solid})\overset{700\;{}^{\circ}C,\;\;{\;N}_{2},\;\;\;3h}{\to }\;{\mathrm{Co}}_{9}S_{8}/\mathrm{CNF}$$

(5) $${\mathrm{Co}}_{9}\;S_{8}/\mathrm{CNF}+{Ni}^{2+}+{2\;Co}^{2+}+{4\;S}^{2-}\overset{Hydrothermal,\;\;\;150\;{}^{\circ}C,\;\;\;8h}{\to }\;{\mathrm{NiCo}}_{2}S_{4}/{\mathrm{Co}}_{9}S_{8}/\mathrm{CNF}$$

### 2.3. Material Characterization

The morphology and microstructure of the samples were examined by field emission scanning electron microscopy (FE-SEM, Hitachi S-4800) and transmission electron microscopy (TEM, Tecnai G2 F20ST Win). Elemental mapping was performed by energy-dispersive X-ray spectroscopy (EDS) coupled with SEM and TEM. X-ray photoelectron spectroscopy (XPS; Thermo Scientific, Waltham, MA, USA, Al Kα radiation, 1486.6 eV) was performed under high-vacuum conditions (5 × 10^−9^ Torr) to analyze the surface chemical states. Crystallographic phases were identified by powder X-ray diffraction (XRD; MPD 3 kW, PANalytical) using Cu Kα radiation (λ = 1.5406 Å) over a 2θ range of 10 to 80° with a step size of 0.02°. Textural properties, including surface area and pore-size distribution, were determined from N_2_ adsorption–desorption isotherms using the Brunauer–Emmett–Teller (BET) method (3-Flex physisorption analyzer, Micromeritics, Norcross, GA, USA).

- Electrochemical measurements

Electrochemical tests were conducted to evaluate the activity, kinetics, and durability of the catalysts in both OER and MOR reactions. All measurements were performed using a Corr Test CS electrochemical workstation in a conventional three-electrode setup. Catalyst-coated nickel foam (1.0 × 0.5 cm^2^) was used as the working electrode, a platinum mesh (1.0 × 1.0 cm^2^) as the counter electrode, and a Hg/HgO electrode as the reference electrode. All experiments were conducted in 1.0 M KOH electrolyte at room temperature. Catalyst inks were prepared by dispersing 10 mg of sample (CS/CNFs, NCS, or NCS/CS/CNFs) in a 1 mL ethanol/deionized water mixture (3:7 *v*/*v*) containing 30 μL of 5 wt% Nafion solution, followed by ultrasonication for 1 h. The suspension was drop-cast onto Ni-foam substrates, and no electrochemical pre-activation was applied.

Potentials were converted to the reversible hydrogen electrode (RHE) scale using the following [37]:

E_RHE_ = E_Hg/HgO_ + 0.924 V (6)

OER activity was measured by linear sweep voltammetry (LSV) at a scan rate of 2 mV/s. Ohmic drop correction was applied according to [37,38]:

E_corr_ = E_measured_ − iR (7)

The overpotential (η) was calculated as follows [13]:

η = E_RHE_ − 1.23 V (8)

Tafel slopes were obtained from LSV data according to [39]:

η = a + b log (j)(9)

where j is the current density and a and b are fitting constants.

Electrochemical impedance spectroscopy (EIS) was conducted over the frequency range of 0.01 Hz to 0.1 MHz to assess charge-transfer resistance. Long-term OER durability was evaluated by chronoamperometry (CA) at 0.65 V (vs. Hg/HgO) for 5 h. In addition, the MOR activity of the samples was studied in 1.0 M KOH with varying concentrations of methanol (0.1 to 1.5 M). A combination of CV, LSV, and CA techniques was employed to comprehensively measure the catalytic performance of the samples toward methanol oxidation.

## 3. Results and Discussion

### 3.1. Surface Morphology and Elemental Analysis

**Figure 2:**
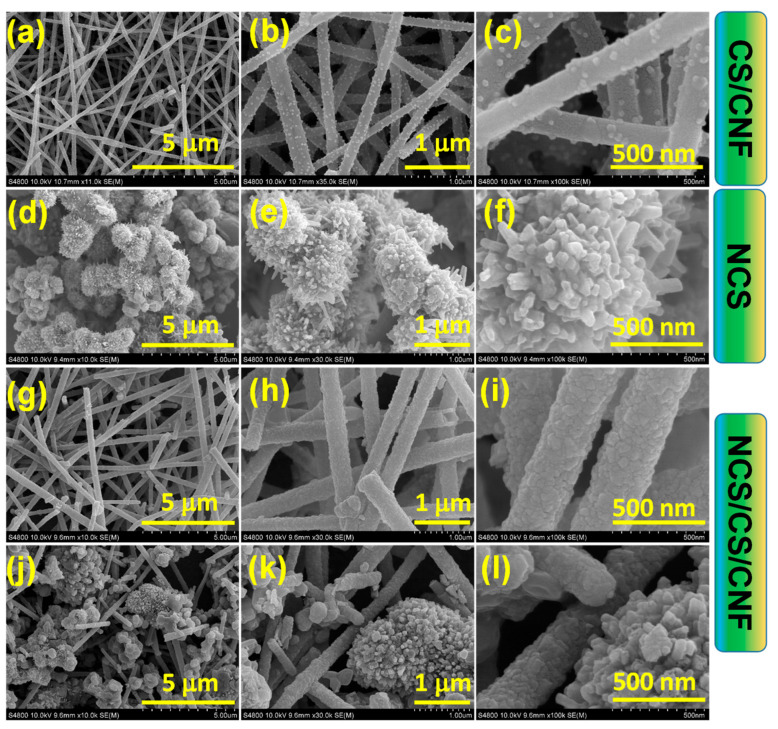
FESEM images of (**a**–**c**) CS/CNFs, (**d**–**f**) NCS, and (**g**–**l**) NCS/CS/CNFs from two distinct regions. Scale bars: 5 μm (**a**,**d**,**g**,**j**), 1 μm (**b**,**e**,**h**,**k**), and 500 nm (**c**,**f**,**i**,**l**).

The surface morphology of the synthesized nanostructures was systematically examined by FESEM, and the results are shown in Figure 2. At low magnification (Figure 2a), the CS/CNF composite exhibits a uniform, three-dimensional nanofibrous network with highly interconnected fibers and abundant void spaces. Such porous fibrous architectures are known to promote rapid ion/electron transport and mechanical robustness in electrochemical systems [40]. At medium magnification (Figure 2b), the nanofiber surfaces are uniformly decorated with fine nanoparticles (~30 nm), indicating successful incorporation/embedding of cobalt sulfide species. The conformal and continuous coating of nanoparticles along the CNF backbone (Figure 2c) provides intimate interfacial contact. It creates a robust platform for further nucleation of active materials, in agreement with previous reports on CNF-supported sulfide systems [41,42]. The morphology of NCS was synthesized without the use of a carbon nanofiber scaffold, as displayed in Figure 2d–f. At a lower magnification (Figure 2d), the NCS sample exhibits aggregated microspheres with rough, flower-like surfaces. Each microsphere consists of densely packed nanosheets, forming a hierarchical, petal-like architecture (Figure 2e). Such ultrathin nanosheets expose a large number of electroactive sites, which are favorable for redox activity (Figure 2f). However, the random aggregation of microspheres may hinder ion diffusion and compromise mechanical stability during extended cycling [43,44]. Figure 2g–l presents the FESEM images of the hierarchical NCS/CS/CNF hybrid composite from two representative regions. At low magnification (Figure 2g), the fibrous morphology of the CNF scaffold remains intact following hydrothermal growth, confirming its role as a mechanically robust host. A closer inspection (Figure 2h) reveals that NiCo_2_S_4_ nanostructures are uniformly anchored onto the CS/CNFs, forming a distinct core-shell configuration. The nanosheet-based shell has an estimated thickness of ~70 ± 5 nm (Supplementary Figure S1a), which increases the overall fiber diameter to ~290 nm ± 30 nm (Figure S1b). Such core–shell architectures are known to enhance electron transport, improve active surface exposure, and accelerate electrolyte diffusion [45,46,47]. High-magnification imaging (Figure 2i) reveals rough and porous shell features, further improving ion accessibility.

The second region (Figure 2i,j) confirms a consistent morphology, where NiCo_2_S_4_ nanosheets retain their flower-like features but are now firmly anchored and evenly dispersed in the composite. This uniform anchoring prevents aggregation, enhances mechanical stability, and establishes conductive pathways along the CNF backbone. Such uniform anchoring of hierarchical sulfides onto conductive supports has been widely recognized as an effective strategy for enhancing electrochemical efficiency [48,49,50].

The elemental distribution of the NCS/CS/CNF hybrid was further examined using energy-dispersive X-ray spectroscopy (EDS) (Figure S2). The elemental maps reveal a homogeneous distribution of Ni, Co, S, and C throughout the nanofibrous framework, with no impurities detected. Quantitative EDS analysis (Figure S2, spectrum, and table) yielded atomic ratios of Ni (11.51 at.%), Co (19.92 at.%), S (30.48 at.%), and C (29.46 at.%), which are consistent with the intended stoichiometry of the NiCo_2_S_4_ phase and the carbon scaffold. The uniform elemental dispersion agrees well with prior studies and supports the robust structural and compositional integrity of the NCS/CS/CNF hybrid [51,52].

**Figure 3:**
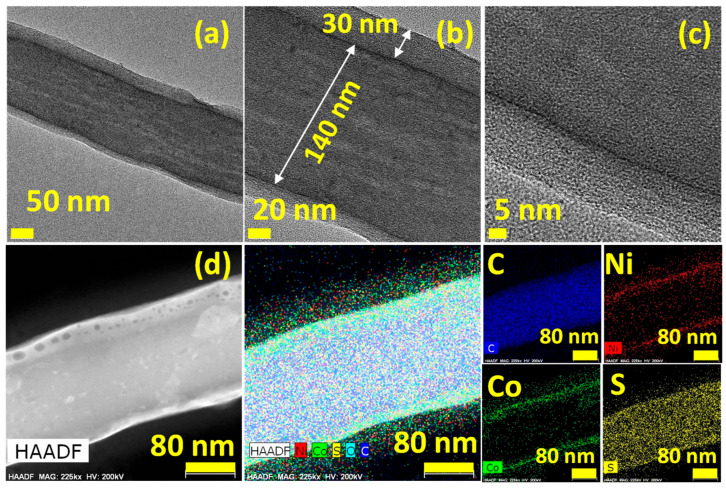
(**a**–**c**) TEM and HRTEM images of NCS/CS/CNFs; (**d**) HAADF image and corresponding elemental maps for C, Ni, Co, and S.

To clarify the core-shell architecture, high-resolution transmission electron microscopy (HRTEM) and high-angle annular dark-field (HAADF) imaging were conducted (Figure 3). The TEM images (Figure 3a–c) clearly show a nanofiber core with a diameter of 140 nm, enveloped by a conformal NiCo_2_S_4_ shell 30 nm thick, which aligns well with the FESEM measurement. This type of NiCo_2_S_4_ core-shell nanostructure has been demonstrated in similar energy storage and conversion systems [28,53,54,55]. Slight variations in shell thickness are attributed to localized differences in nucleation and growth during the hydrothermal process; these are also evident in Figure 3a,b. The HRTEM image (Figure 3c) lacks visible lattice fringes, suggesting that the NiCo_2_S_4_ shell is amorphous or poorly crystalline in this region. Such mixed-phase (amorphous/crystalline) structures are often advantageous in electrochemical contexts, providing abundant active sites, enabling improved ion transport, and offering outstanding structural stability [56,57,58]. HAADF imaging, along with EDS elemental mapping (Figure 3d), further confirms the core–shell morphology. The analysis reveals that the shell primarily concentrates Ni, Co, and S, while the fiber core mainly confines carbon. The distribution appears more even, extending across both regions and suggesting either partial interdiffusion or uniform surface coverage. This imaging pattern is reinforced by the line-scan elemental profile (Figure S3), where the intensities of Ni, Co, and S rise sharply at the fiber edges, contrasting with the uniform distribution of carbon throughout the cross-section. Collectively, these findings validate the successful formation of the NCS/CS/CNF core-shell structure. This design leverages the high electrical conductivity of the carbon core together with the redox-rich sulfide shell, offering a synergistic strategy for enhanced electrochemical performance [59,60].

### 3.2. Structural and Surface Chemistry Analysis

**Figure 4:**
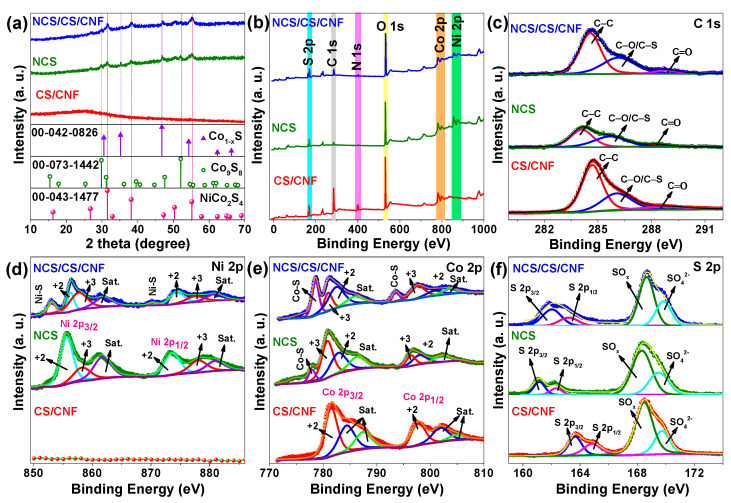
(**a**) XRD spectra; (**b**) XPS survey spectra; and (**c**) C 1s, (**d**) Ni 2p, (**e**) Co 2p, and (**f**) S 2p XPS spectra of CS/CNF, NCS, and NCS/CS/CNF samples.

The structural and surface chemistry characteristics of the CS/CNF, NCS, and NCS/CS/CNF composites were systematically analyzed using XRD and XPS, as presented in Figure 4. The XRD pattern of CS/CNFs (Figure 4a) shows a broad hump without any distinct diffraction peaks, confirming its amorphous nature. This feature is typical of carbon nanofiber-based frameworks that lack long-range crystallinity [61]. Importantly, no crystalline reflections corresponding to cobalt sulfide were detected, suggesting that cobalt species exist either in disordered domains or as highly dispersed nanoclusters embedded in the CNF framework. This assumption was further supported by elemental mapping and EDS analysis (Figure S4), which revealed the uniform distribution of C, S, and Co throughout the fibrous network. Quantitative EDS confirmed the incorporation of 46.73 wt% C, 16.48 wt% S, and 13.37 wt% Co, thereby validating the successful doping of cobalt and sulfur into the CNF structure despite the absence of crystalline phases in XRD.

In contrast, the NCS sample displays well-defined peaks at 2θ values of 26.8°, 31.5°, 38.1°, 50.2°, and 55.3°, which are indexed to the (220), (311), (222), (400), (422), (511), and (440) planes of the spinel NiCo_2_S_4_ phase (JCPDS No. 00-043-1477) [62,63]. Additional reflections corresponding to Co_9_S_8_ (JCPDS No. 01-073-1442) and Co_1-x_S (JCPDS No. 00-042-0826) can also be observed, suggesting the coexistence of secondary cobalt sulfide phases, possibly due to local stoichiometric inhomogeneity or kinetically limited nucleation during synthesis [64,65,66]. The NCS/CS/CNF composite exhibits a hybrid XRD profile, where the crystalline reflections of NiCo_2_S_4_ are clearly retained alongside the broad amorphous hump of CNFs. This coexistence of crystalline NiCo_2_S_4_ with an amorphous CNF framework demonstrates the successful integration of active sulfide nanostructures into a conductive carbon scaffold. Such hybridization is advantageous because it combines the fast electron transport pathways of CNFs with the multi-redox activity of sulfides, thereby promoting synergistic electrochemical behavior effects [67].

The XPS survey spectra (Figure 4b) further corroborate these findings, showing characteristic signals for C 1s, O 1s, N 1s, S 2p, Ni 2p, and Co 2p in both NCS and NCS/CS/CNF samples. In contrast, CS/CNFs lack Ni-related peaks, consistent with the absence of nickel precursors. The relative intensity of C 1s and O 1s peaks in CS/CNFs and NCS/CS/CNFs suggests the presence of abundant oxygenated functional groups on CNFs, which may facilitate strong interfacial bonding with transition metal sulfides. High-resolution C 1s spectra (Figure 4c) deconvolute into three main components: (i) a peak at 284.6–284.7 eV (C-C/C=C, sp^2^-hybridized graphitic carbon), (ii) a peak at 258.8–286.2 eV (C-O/C-S bonds; hydroxyl, ether, or thiol groups), and (iii) a peak at 288.4–288.8 eV (C=O or O-C=O, carbonyl or carboxyl functionalities) [68,69,70]. Among the samples, NCS/CS/CNFs exhibit the strongest C-O/C-S and C=O features, suggesting enhanced surface oxidation and strong interfacial coupling between sulfides and CNFs, which are essential for enhancing charge transfer efficiency.

The Ni 2p spectra (Figure 4d) provide detailed information on the oxidation states and chemical environment of nickel in the composite systems. For the NCS sample, the Ni 2p_3/2_ and Ni 2p_1/2_ peaks at 855.4 eV and 873.6 eV correspond to Ni^2+^ species in sulfide phases, such as the NiCo_2_S_4_ phase [71,72]. Additional peaks at 858.3 eV and 877.9 eV can be attributed to Ni^3+^ species, indicating a mixed-valence Ni^2+^/Ni^3+^ couple. The accompanying satellite peaks at 861.71 eV and 881.1 eV arise from shake-up transitions of high-spin Ni^2+^. Remarkably, in the NCS/CS/CNF composite, a chemical shift toward lower binding energies can be observed, with peaks at 853.1 eV (2p_3/2_) and 870.4 eV (2p_1/2_) assigned to Ni-S covalent bonds [73]. This shift indicates stronger Ni-S interactions and enhanced electronic coupling with CNFs. Additional contributions at 856.3 eV, 857.9 eV, 874.5 eV, and 877.5 eV, along with satellite peaks at 861.9 eV and 880.9 eV, suggest the continued coexistence of Ni^2+^/Ni^3+^ states. These results suggest that electron transfer between NiCo_2_S_4_ and the conductive CS/CNF matrix results in electronic modulation, thereby enhancing the delocalization of charge and stabilizing sulfide species within the CNFs.

The Co 2p spectra (Figure 4e) reveal distinct differences in the chemical states of the cobalt among all samples. In the CS/CNF sample, two prominent peaks can be observed at 781.4 eV (Co 2p_3/2_) and 797.7 eV (Co 2p_1/2_), along with satellite features at 784.6, 787.6, 802, and 804.9 eV [73]. These binding energies are characteristic of Co^2+^ in Co_9_S_8_, with strong shake-up satellites resulting from high-spin Co^2+^ in an octahedral Co-S coordination environment [74]. The absence of Co^0^ or Co^3+^ contributions indicates that cobalt exists almost exclusively in the +2 oxidation state in CS/CNFs, consistent with a stable Co_9_S_8_ phase embedded within the CNF matrix [75]. In contrast, the NCS sample exhibits a more complex Co 2p spectrum, with Co 2p_3/2_ peaks at 777.9, 780.9, and 782.9 and Co 2p_1/2_ peaks at 796.4, 798.2, and 802.4 eV [73,76]. The presence of multiple components and satellite features at ~786.5 eV confirms the existence of Co^2+^ and Co^3+^ oxidation states, a characteristic known to be present in spinel-structured NiCo_2_S_4_. These findings are in close agreement with previous studies, which have shown that Co^2+^ and Co^3+^ coexist due to the mixed-valence occupation of tetrahedral and octahedral Co sites in the spinel lattice, contributing to enhanced redox activity. For the NCS/CS/CNF composite, the Co 2p_3/2_ spectrum includes peaks at 778.6, 780.9, and 782.7 eV, while the Co 2p_1/2_ region displays peaks at 793.6, 797.7, 801.1, and 803.7 eV. The low-binding energy feature at 778.6 is attributed to metallic Co^0^, suggesting the presence of partially reduced cobalt species, likely due to interfacial electronic interactions with the conductive CNF framework. The simultaneous presence of Co^0^, Co^2+^, and Co^3+^ in NCS/CS/CNFs indicates a broader distribution of cobalt oxidation states than in either CS/CNFs or NCS alone. This expanded redox window can be ascribed to the synergistic effect of phase integration and the conductive CNF matrix, which may facilitate electron delocalization and structural flexibility. Moreover, the broadened and intensified satellite peaks suggest strong Co-S covalence and charge transfer interactions, contributing to the improved electrochemical performance observed in the composite system.

The S 2p high-resolution XPS spectra (Figure 4f) were deconvoluted into S 2p_3/2_ and S 2p_1/2_ spin-orbit doublets with a consistent energy separation of ~1.18 eV and a fixed 2:1 area ratio, as expected for sulfur species. In the CS/CNF sample, two peaks centered at 163.65 eV (S 2p_3/2_) and 164.83 eV (S 2p_1/2_) are attributed to disulfide (S_2_^2−^) or C-S bonding, which are typically observed in carbon-supported cobalt sulfides [77,78,79]. Peaks at 168.35 (S 2p_3/2_) and 169.53 eV (S 2p_1/2_) correspond to oxidized sulfur species (e.g., SO_x_^−^ and SO_4_^2^^−^) formed upon exposure to air. In the NCS sample, the prominent peaks at 161.35 eV (S 2p_3/2_) and 162.53 eV (S 2p_1/2_) are attributed to S^2−^ species in metal-sulfide bonds, specifically Ni-S and Co-S coordination within the NiCo_2_S_4_ lattice. Additionally, distinct oxidized sulfur peaks can be observed at 168.53 eV (S 2p_3/2_) and 169.71 eV (S 2p_1/2_), confirming the coexistence of reduced and oxidized sulfur states in NCS. For the NCS/CS/CNF composite, two well-defined doublets appear at 162.03/163.21 eV (S 2p_3/2_/S 2p_1/2_) and 168.66/169.84 eV, representing reduced and oxidized sulfur, respectively. The relative proportion of oxidized sulfur (~71%) indicates enhanced surface oxidation and charge redistribution at the NCS-carbon interface, consistent with strong interfacial coupling between NiCo_2_S_4_, Co_9_S_8_, and the CNF matrix [53,77,80,81,82]. Overall, the XPS results clearly demonstrate that all samples contain both reduced and oxidized sulfur species, with the oxidized fraction increasing systematically from NCS < NCS/CS/CNFs < CS/CNFs. These XPS findings, in conjunction with XRD analysis, confirm that the CS/CNF sample consists of amorphous carbon nanofiber with oxidized Co species and covalently bonded sulfur. Also, the NCS and NCS/CS/CNF samples exhibit crystalline NiCo_2_S_4_ and Co_9_S_8_ phases featuring mixed-valence sulfur environments and strong electronic interactions at the sulfide-carbon interface.

The high-resolution O 1s XPS spectra of the CS/CNF, NCS, and NCS/CS/CNF samples (Figure S5) were deconvoluted into three distinct components, denoted as O_I_, O_II_, and O_III_, corresponding to different oxygen chemical states. The first peak (O_I_, ≈530.1–530.3 eV) originates from lattice oxygen (O^2−^) bound to metal cations such as Ni or Co in metal-oxygen or oxy-sulfide bonds [67,72]. The second peak (O_II_, ≈531.4–531.7 eV) is assigned to oxygen in surface hydroxyl groups (-OH) or oxygen-vacancy-related species, indicating structural defects and unsaturated coordination sites that enhance electronic conductivity and redox activity [72,75]. The third component (O_III_, ≈532.6–533.1 eV) corresponds to adsorbed oxygen species or oxygenated surface groups (C-O, C=O, or H_2_O) arising from partial surface oxidation of carbon or metal sulfides [75,77]. Among the samples, CS/CNFs exhibit the strongest O_III_ contribution, reflecting the abundance of oxygen-containing functional groups on the CNF surfaces [75,77]. NCS exhibits a dominant O_I_ peak, consistent with lattice oxygen within the NiCo_2_S_4_ framework [67,72]. In contrast, the NCS/CS/CNF composite exhibits enhanced O_I_ and O_II_ features, indicating the presence of lattice oxygen and surface hydroxyl/defect content at the NCS-carbon interface. These findings reveal that the hybrid structure possesses more chemically diverse and defect-rich oxygen species, which facilitate charge transfer and ion transport. This oxygen-rich and defect-engineered surface promotes enhanced OH^^−^^ adsorption and lattice oxygen participation during redox reactions, thereby improving the electrochemical performance of the NCS/CS/CNF composite [75,83].

Figure S6 presents the nitrogen (N_2_) adsorption-desorption measurements and pore-size distributions of the CS/CNF, NCS, and NCS/CS/CNF samples. Figure S6a–c display the adsorption-desorption isotherms recorded at 77 K. The progressive increase in adsorption and the more pronounced hysteresis observed for NCS/CS/CNFs indicate the formation of a more open mesoporous framework with a larger accessible pore volume compared with the CS/CNF and NCS samples. The BET surface areas of the samples were measured as 6.12 m^2^/g for CS/CNFs, 4.85 m^2^/g for NCS, and 24.84 m^2^/g for NCS/CS/CNFs. Figure S6d–f illustrate the BJH pore-size distributions derived from the desorption branches. CS/CNFs exhibit a narrow mesopore distribution centered at 6.50 nm, with a pore volume of 0.0080 cm^3^/g. NCS displays a broader distribution centered at 14.5 nm, with a pore volume of 0.0173 cm^3^/g. In contrast, NCS/CS/CNFs demonstrate a dominant mesopore centered at 8.29 nm, along with the highest pore volume (0.0424 cm^3^/g). Overall, the NCS/CS/CNF composite provides a hierarchical mesoporous architecture that enhances the electrolyte-accessible surface area and pore volume, which is expected to improve ion transport and electrochemical accessibility.

### 3.3. Electrocatalytic Activity for OER

**Figure 5:**
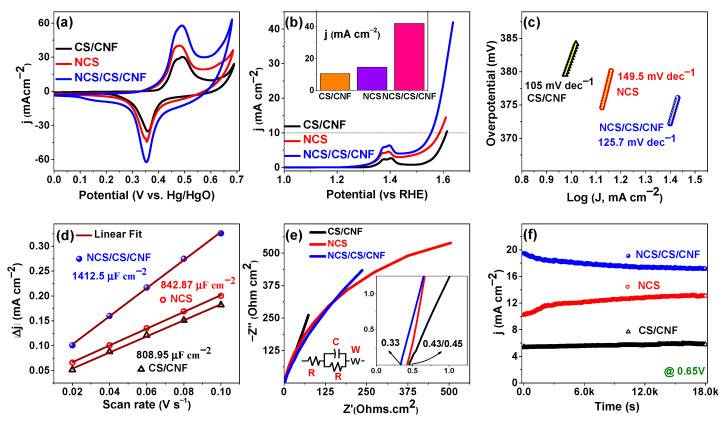
OER performance of CS/CNF, NCS, and NCS/CS/CNF electrodes in 1 M KOH: (**a**) CV curves at 20 mV s^−1^, (**b**) LSV profiles, (**c**) Tafel slopes, (**d**) areal capacitance derived from CV, (**e**) Nyquist plots with equivalent circuit model, and (**f**) chronoamperometry stability at 0.65 V for 5 h.

The electrochemical behavior of CS/CNF, NCS, and NCS/CS/CNF electrodes was systematically examined in a N_2_-saturated 1 M KOH electrolyte using CV, LSV, Tafel analysis, EIS, and CA. As shown in the CV curves at 20 mV/s (Figure 5a), the CS/CNF electrode delivered only weak current responses due to the low conductivity of Co_9_S_8_ and the electrochemical inertness of CNFs. In contrast, NCS exhibited well-defined redox peaks with significantly higher current density, confirming enhanced Faradaic activity from the Ni/Co redox centers and the conductive sulfide framework. Remarkably, the NCS/CS/CNF hybrid electrode achieved the largest CV integral area with pronounced redox features, reflecting high reversibility and efficient utilization of active sites. This enhancement arises from the synergistic contributions of the conductive CNFs, electroactive NCS, and the structural stability imparted by the CS/CNF framework [84,85].

The LSV profiles (Figure 5b) further highlight the catalytic differences. CS/CNFs required a high overpotential of 382 mV to achieve a current density of 10 mA cm^−2^, while NCS reduced the overpotential to 355 mV due to its abundant redox-active sites. Notably, the NCS/CS/CNF hybrid achieved the lowest overpotential (324 mV) with the highest current density, indicating accelerated charge-transfer kinetics and enhanced catalytic efficiency. Consistent with these results, Tafel slope (Figure 5c) analysis revealed sluggish kinetics for CS/CNFs (105.2 mV dec^−1^), moderate improvement for NCS (149.52 mV dec^−1^), and the most favorable slope for NCS/CS/CNFs (125.8 mV dec^−1^), confirming efficient electron transport in the hybrid structure [86,87].

The electrochemically active surface area (ECSA) was estimated from cyclic voltammetry (CV) performed in the non-Faradaic region (0.10–0.20 vs. Hg/HgO) at scan rates of 20 to 100 mV/s (Figure S7). The slope of the current density difference versus the scan rate graph was used to determine the double-layer capacitance (C_dl_), which is proportional to the ECSA [88]. Capacitance (C_dl_) measurements (Figure 5d) derived from CV curves demonstrated that NCS/CS/CNFs delivered the highest areal capacitance (1412.5 μF cm^−2^), outperforming both NCS (842.87 μF cm^−2^) and CS/CNFs (808.95 μF cm^−2^). The larger ECSA of the hybrid electrode (35.31 cm^2^) compared to NCS (21.07 cm^2^) and CS/CNFs (20.22 cm^2^) underscores its superior accessibility of electrochemically active sites and more efficient ion transport. EIS analysis (Figure 5e) further corroborates these results. The Nyquist plots exhibit a depressed semicircle in the high-to-medium frequency region, indicative of non-ideal capacitive behavior and surface heterogeneities. The intersection of the plots on the real impedance axis at higher frequencies represents the solution resistance (R_s_). The measured R_s_ values for CS/CNFs, NCS, and NCS/CS/CNFs were found to be 0.33, 0.43, and 0.45 Ohm cm^2^, respectively. The smaller R_s_ and reduced charge-transfer resistance (R_ct_) of the NCS/CS/CNF electrode reveal faster electron and ion transport at the electrode–electrolyte interface [89,90]. These results demonstrate that the hybrid architecture provides abundant active sites and highly conductive interfaces, promoting rapid charge transfer and improved reaction kinetics, consistent with previous findings for NiOOH-CuO nano-heterostructures, where synergistic electronic interactions enhanced both OER and ethanol oxidation performance [88].

The stability (Figure 5f) of all the prepared samples was evaluated using CA at 0.65 V for 5 h. All electrodes maintained relatively stable currents with minimal decay, but the NCS/CS/CNF electrode exhibited the most robust performance. This durability can be attributed to the conductive CNF backbone, the structural reinforcement from CS, and the chemical stability of NiCo_2_S_4_ under alkaline conditions. Overall, the NCS/CS/CNF electrode demonstrated excellent OER activity, stability, and durability, making it a promising candidate for long-term electrochemical energy conversion.

### 3.4. Methanol Oxidation Reaction (MOR) Performance as a Bifunctional Extension

**Figure 6:**
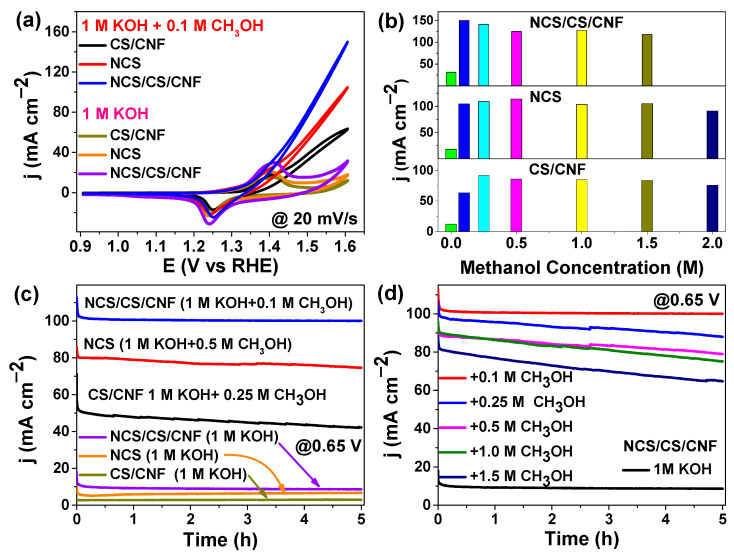
MOR performance of CS/CNF, NCS, and NCS/CS/CNF electrodes: (**a**) CV curves in 1 M KOH with and without methanol at 20 mV s^−1^; (**b**) comparison of anodic current densities at different methanol concentrations; (**c**) chronoamperometry stability curves of CS/CNFs (1 M KOH with/with 0.25 M CH_3_OH), NCS (1 M KOH with/with 0.5 M CH_3_OH), and NCS/CS/CNFs (1 M KOH with/with 0.1 M CH_3_OH) at 0.65 V for 18,000 s; and (**d**) long-term durability tests of NCS/CS/CNFs at various methanol concentrations in 1 M KOH.

To further explore the multifunctional catalytic behavior of the designed materials, the methanol oxidation activities of CS/CNF, NCS, and NCS/CS/CNF electrodes were evaluated in N_2_-saturated 1.0 M KOH using CV and CA. As shown in Figure 6a, all catalysts exhibit characteristic Ni/Co redox peaks in 1.0 M KOH at a scan rate of 20 mV s^−1^, confirming their intrinsic electrochemical activity. Upon the introduction of methanol (0.1–2 M), the anodic current density increases significantly for all electrodes, reflecting active methanol electro-oxidation at the Ni/Co active sites. Among the samples, the NCS/CS/CNF electrode delivers the highest current response (Figure 6b), achieving 150 mA/cm^2^ at just 0.1 M methanol, while CS/CNF and NCS reach 91.6 and 114 mA cm^−2^ at 0.25 M and 0.5 M methanol, respectively. These results indicate that NCS/CS/CNFs can efficiently catalyze methanol oxidation even at low methanol concentrations, highlighting their superior bifunctional catalytic activity.

The methanol oxidation on the NCS/CS/CNF catalyst proceeds through a multi-step reaction mechanism involving surface adsorption, intermediate oxidation, and electron transfer, as described by the following reactions:

(10) $$N/\mathrm{Co}\;+\;2{OH}^{-}\to \;\mathrm{Ni}(\mathrm{OH}{)}_{2}/\mathrm{Co}(\mathrm{OH}{)}_{2}+2e^{-}$$

(CH_3_OH)_bulk_ → (CH_3_OH)_surface_ (diffusion) (11)

(CH_3_OH)_surface_ → (CH_3_OH)_ads_ (adsorption) (12)

(13) $$\mathrm{Ni}(\mathrm{OH}{)}_{2}/\mathrm{Co}(\mathrm{OH}{)}_{2}+2{OH}^{-}\to \;\mathrm{NiO}(\mathrm{OH})/\mathrm{Co}(\mathrm{OH})+H_{2}O+2e^{-}$$

The NCS/CS/CNF catalyst exhibits a prominent anodic peak at 1.4 V vs. RHE, suggesting improved oxidation kinetics. Furthermore, its onset potential shows a noticeable negative shift compared to CS/CNFs and NCS, implying enhanced methanol adsorption and activation facilitated by synergistic Ni/Co interactions. Quantitatively, the anodic peak current density of NCS/CS/CNFs is approximately 1.63-times and 1.31-times higher than those of CS/CNFs and NCS, respectively, confirming its superior MOR activity.

Long-term stability tests were performed under optimized methanol concentrations corresponding to the highest observed current densities, as shown in Figure 6c. The CA curves recorded at 0.65 V for 18,000 s (5 h) reveal that NCS/CS/CNFs maintain a more stable current response than CS/CNFs and NCS in both KOH and KOH–methanol solutions. Additionally, Figure 6d presents the durability of NCS/CS/CNFs under varying methanol concentrations. The catalyst exhibits the most stable performance in 1 M KOH + 0.1 M CH_3_OH, indicating excellent tolerance to intermediate poisoning and long-term operational stability. Overall, this bifunctional electrocatalyst demonstrates high activity and durability toward both OER and MOR, making it a promising candidate for integrated electrochemical energy conversion systems.

## 4. Conclusions

In conclusion, transition metal sulfides (TMSs) are attractive electrocatalysts because of their tunable electronic structures, natural abundance, and strong interactions with reaction intermediates. In this work, we demonstrated a 3D heterostructure consisting of crystalline NiCo_2_S_4_ grown on amorphous Co_9_S_8_-embedded CNFs, synthesized via a combined electrospinning and hydrothermal route. XRD confirmed the coexistence of spinel NiCo_2_S_4_ and Co_9_S_8_ phases, while FESEM revealed a porous, interconnected network that facilitates efficient ion and mass transport. XPS further verified the presence of multivalent Ni^2+^/Ni^3+^ and Co^2+^/Co^3+^ states, indicating strong electronic interactions and charge redistribution across the heterointerface. The hierarchical architecture integrates three functional components: (i) a conductive CNF backbone for rapid electron transport and mechanical robustness, (ii) amorphous Co_9_S_8_ domains that introduce defect-rich active sites that accelerate reaction kinetics, and (iii) a crystalline NiCo_2_S_4_ shell that enhances multivalent redox activity and provides an abundant electroactive surface area.

As a result of these synergistic features, the NiCo_2_S_4_/Co_9_S_8_-CNF heterostructure exhibits outstanding bifunctional electrocatalytic activity. For the OER, the catalyst delivers a low overpotential of 324 mV at 10 mA cm^−2^, with a Tafel slope of 125.7 mV dec^−1^. In contrast, for the MOR, it achieves a high current density of 150 mA cm^−2^ at 0.1 M methanol and exhibits excellent durability at 0.65 V over 18,000 s. It demonstrates that engineering amorphous/crystalline heterointerfaces provides a practical and scalable strategy for developing next-generation electrocatalysts for alkaline water splitting and direct methanol fuel cells.

## Data Availability

The raw data supporting the conclusions of this article will be made available by the authors on request.

## Supplementary Materials

The following supporting information can be downloaded at https://www.mdpi.com/article/10.3390/nano15201559/s1. Figure S1: (a–b). FESEM images of NCS/CS/CNFs at different magnifications. Figure S2: (Top) SEM image and EDS elemental maps of Ni, Co, C, O, and S. (Bottom) EDS spectrum and quantified elemental composition of the NCS/CS/CNF composite. Figure S3: EDS line-scan profile across a single NCS/CS/CNF. The corresponding HAADF image shows the line-scan region. Figure S4: (a–f) SEM image and EDS elemental maps of C, Co, S, N, and O. (g and h) EDS spectrum and quantified elemental composition of the CS/CNF composite. Figure S5: Deconvoluted O 1s XPS spectra of CS/CNF, NCS, and NCS/CS/CNF samples showing the relative contributions of lattice oxygen, oxygen vacancies, and surface-adsorbed species. Figure S6: N2 adsorption–desorption isotherms (a–c) and BJH pore-size distributions (d–f) for (a,d) CS/CNFs, (b,e) NCS, and (c,f) NCS/CS/CNFs. Figure S7: CV curves of (a) CS/CNFs, (b) NCS, and (c) NCS/CS/CNFs at different scan rates (20, 40, 60, 80, and 100 mV/s) in the potential range of 0.10–0.20 V vs. Hg/HgO, which were used to calculate the Cdl.

## Abbreviations

The following abbreviations are used in this manuscript:

OER	Oxygen evolution reaction
MOR	Methanol oxidation reaction
DMFCs	Direct methanol fuel cells
ORR	Oxygen reduction reaction
NCS/CS/CNFs	NiCo_2_S_4_ grown/anchored on Co_9_S_8_-loaded carbon nanofibers
CS/CNF	Carbon nanofiber
NCS	NiCo_2_S_4_
DI	Deionized
KOH	Potassium hydroxide
PAN	Polyacrylonitrile
DMF	N, N-dimethylformamide
NMP	N-Methyl-2-pyrrolidone
PVDF	polyvinylidene fluoride
TMS	Transition metal sulfide
FESEM	Field emission scanning electron microscopy
TEM	Transmission electron microscopy
XPS	X-ray photoelectron spectroscopy
XRD	Powder X-ray diffraction
BET	Brunauer–Emmett–Teller
RHE	Reversible hydrogen electrode
LSV	Linear sweep voltammetry
ECSA	Electrochemically active surface area
CV	Cyclic voltammetry
EIS	Electrochemical impedance spectroscopy
CA	Chronoamperometry
HAADF	High-angle annular dark-field
