# Supplementary material for: Engineering 3D Heterostructured NiCo2S4/Co9S8-CNFs via Electrospinning and Hydrothermal Strategies for Efficient Bifunctional Energy Conversion
Source: Nanomaterials (Basel). 2025 Oct 13;15(20):1559. doi: 10.3390/nano15201559 (PMC12566664; doi:10.3390/nano15201559)
Supplement: Supplementary file 1 [file nanomaterials-15-01559-s001.zip › nanomaterials-3922988-supplementary.pdf]

nanomaterials

Supplementary Materials

Engineering 3D Heterostructured $NiCo_2S_4/Co_9S_8$-CNFs via Electrospinning and Hydrothermal Strategies for Efficient Bifunctional Energy Conversion

Dhananjaya Merum, Rama Krishna Chava * and Misook Kang *

Department of Chemistry, College of Natural Sciences, Yeungnam University, 280 Daehak-Ro, Gyeongsan, Gyeongbuk 38541, Republic of Korea; msdhana@yu.ac.kr (D.M.)
* Correspondence: mskang@ynu.ac.kr (M.K.); rama@ynu.ac.kr (R.K.C.)

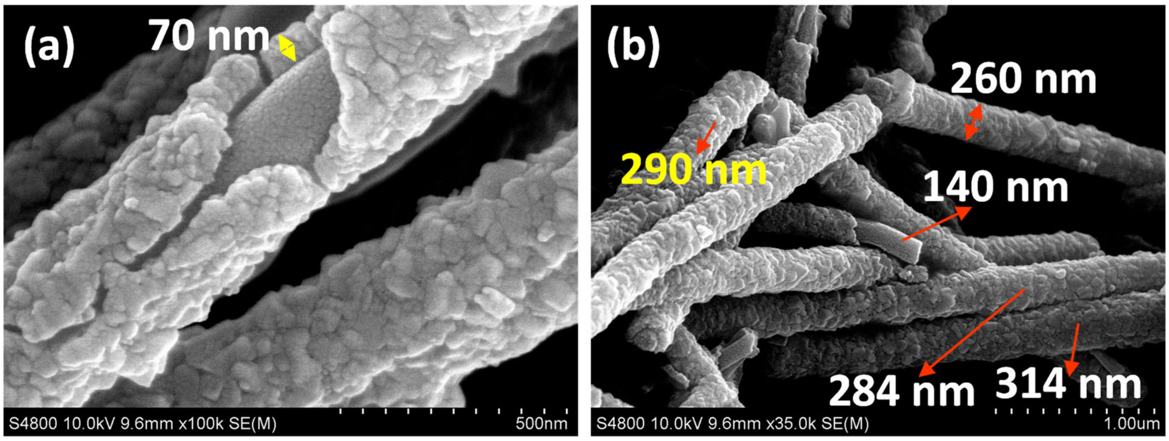

Figure S1. (a–b). FESEM images of NCS/CS/CNFs at different magnifications.

 https://doi.org/10.3390/nano15201559

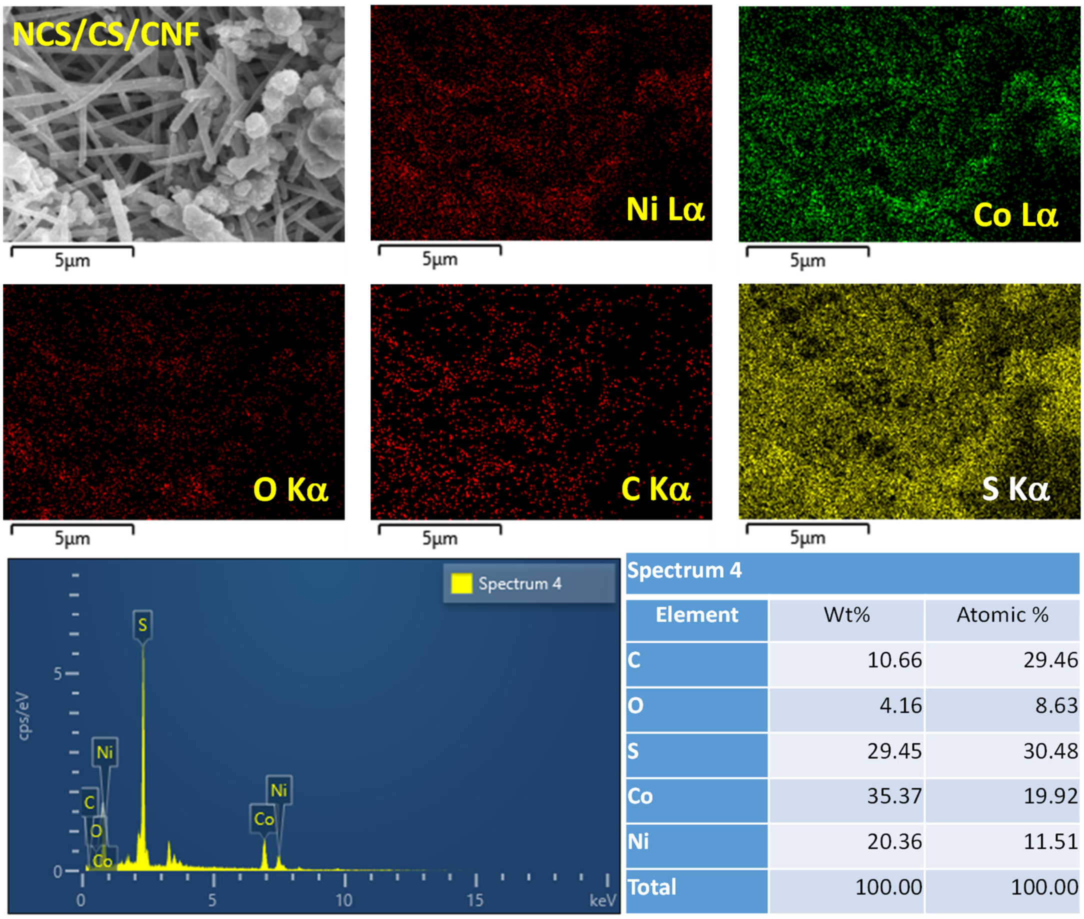

Spectrum 4		
Element	Wt%	Atomic %
C	10.66	29.46
O	4.16	8.63
S	29.45	30.48
Co	35.37	19.92
Ni	20.36	11.51
Total	100.00	100.00

Figure S2. (Top) SEM image and EDS elemental maps of Ni, Co, C, O, and S. (Bottom) EDS spectrum and quantified elemental composition of the NCS/CS/CNF composite.

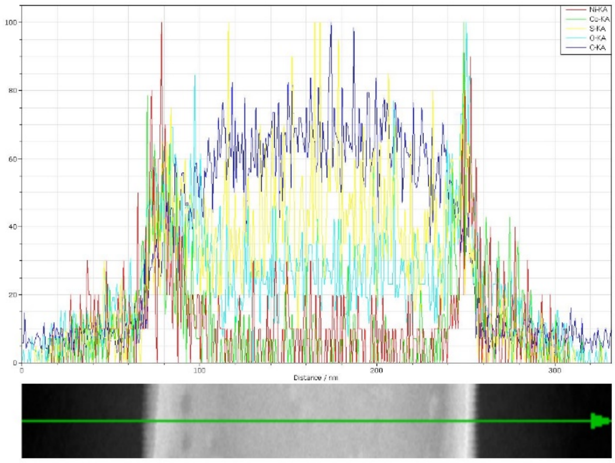

Figure S3. EDS line-scan profile across a single NCS/CS/CNF. The corresponding HAADF image shows the line-scan region.

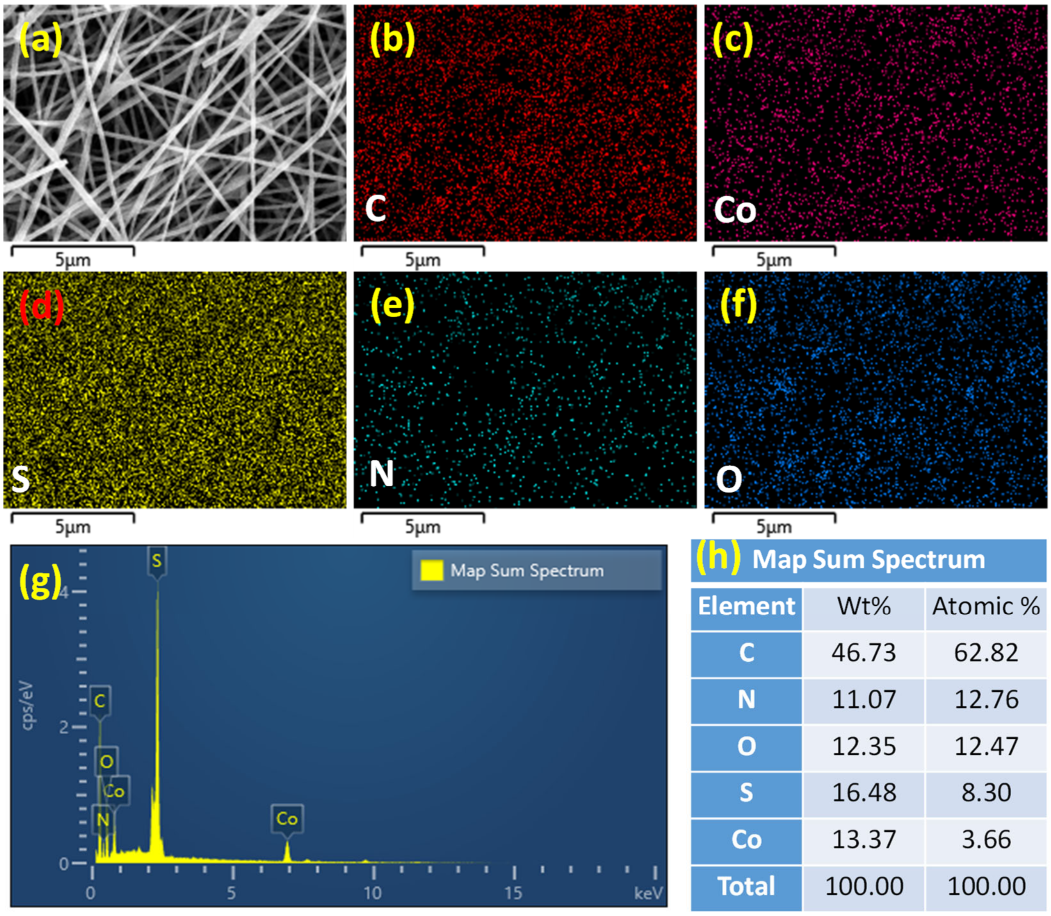

Element	Wt%	Atomic %
C	46.73	62.82
N	11.07	12.76
O	12.35	12.47
S	16.48	8.30
Co	13.37	3.66
Total	100.00	100.00

Figure S4. (a–f) SEM image and EDS elemental maps of C, Co, S, N, and O. (g and h) EDS spectrum and quantified elemental composition of the CS/CNF composite.

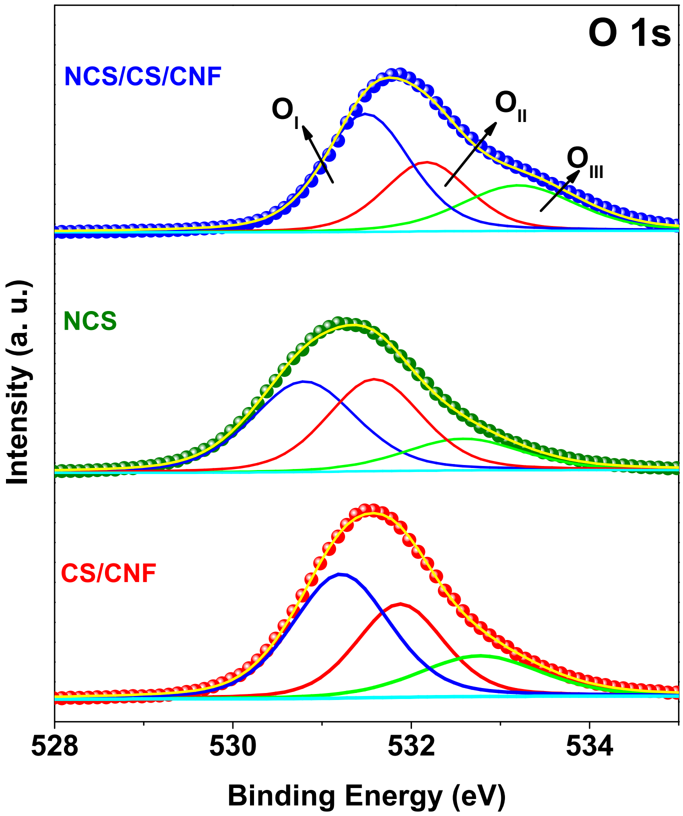

Figure S5. Deconvoluted O 1s XPS spectra of CS/CNF, NCS, and NCS/CS/CNF samples showing the relative contributions of lattice oxygen, oxygen vacancies, and surface-adsorbed species.

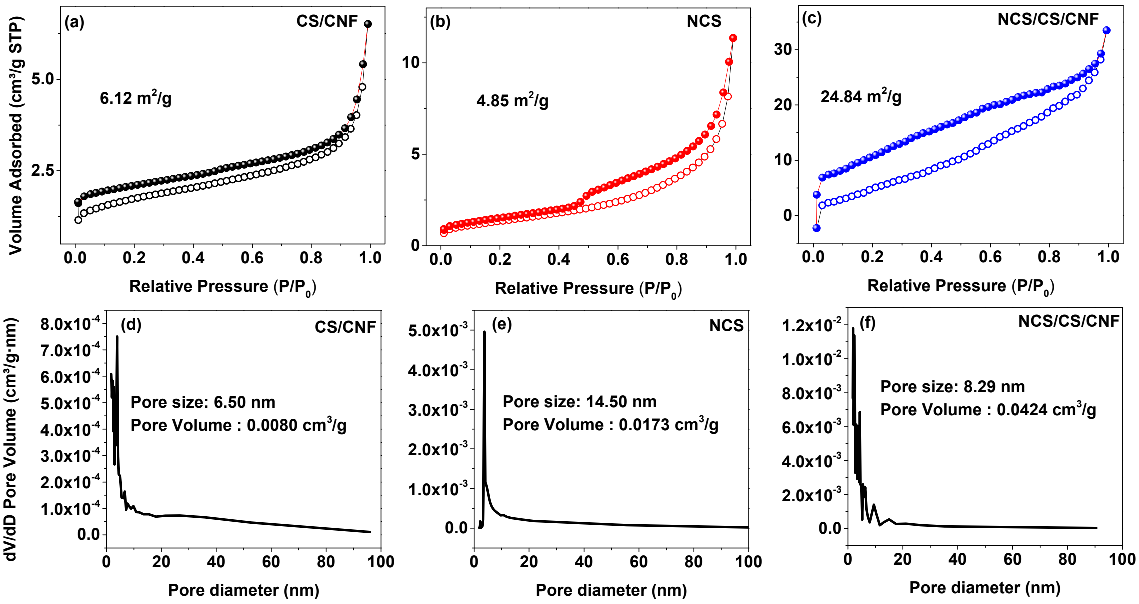

Figure S6. N_2 adsorption–desorption isotherms (a–c) and BJH pore-size distributions (d–f) for (a,d) CS/CNFs, (b,e) NCS, and (c,f) NCS/CS/CNFs.

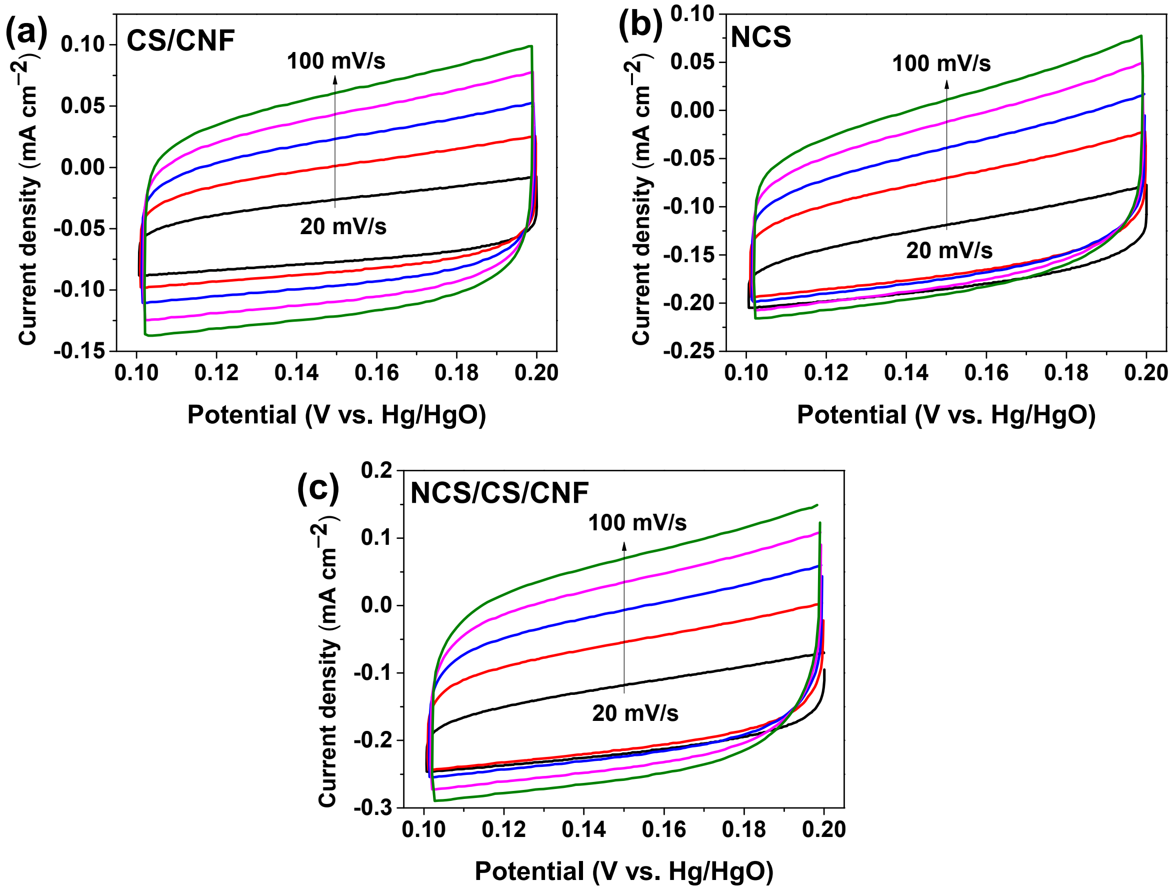

Figure S7. CV curves of (a) CS/CNFs, (b) NCS, and (c) NCS/CS/CNFs at different scan rates (20, 40, 60, 80, and 100 mV/s) in the potential range of 0.10–0.20 V vs. Hg/HgO, which were used to calculate the C_{dl}.

Disclaimer/Publisher's Note: *The statements, opinions and data contained in all publications are solely those of the individual author(s) and contributor(s) and not of MDPI and/or the editor(s). MDPI and/or the editor(s) disclaim responsibility for any injury to people or property resulting from any ideas, methods, instructions or products referred to in the content.*
